# The landscape of recurrent spontaneous abortion registered on clinical trials.gov

**DOI:** 10.3389/fendo.2024.1460968

**Published:** 2024-12-20

**Authors:** Xiaoling Zhou, Fan Lai, Wei Chen, Congrong Zhou, Yi Deng, Tao Wang, Shasha Xing, Haoyang Diao, Mi Tang, Wenmei Guo, Erdan Luo

**Affiliations:** ^1^ Department of Pharmacy, Chengdu Women’s and Children’s Central Hospital, School of Medicine, University of Electronic Science and Technology of China, Chengdu, China; ^2^ Department of Obstetrics, Chengdu Women’s and Children’s Central Hospital, School of Medicine, University of Electronic Science and Technology of China, Chengdu, China; ^3^ Department of Traditional Chinese Medicine, Chengdu Women’s and Children’s Central Hospital, School of Medicine, University of Electronic Science and Technology of China, Chengdu, China; ^4^ Department of Good Clinical Practice, Chengdu Women’s and Children’s Central Hospital, School of Medicine, University of Electronic Science and Technology of China, Chengdu, China

**Keywords:** clinical trials, recurrent spontaneous abortion, recurrent pregnancy loss, recurrent miscarriage, habitual abortion

## Abstract

**Objective:**

Recurrent spontaneous abortion (RSA) presents a significant challenge in the field of reproductive medicine, as effective treatments remain limited despite extensive research efforts. A comprehensive understanding of current RSA clinical trials is essential for enhancing trial design and identifying existing research gaps. The aim of this study is to characterize RSA related clinical trials registered on Clinical Trials.gov.

**Methods:**

A thorough search was conducted to identify and review clinical trials focusing on RSA that were registered on Clinical Trials.gov up to March2, 2024.

**Results:**

A total of 138 trials were identified in the analysis, with 72 (52.17%) classified as intervention trials and 66 (47.83%) as observational trials. Approximately half of the studies (67,48.55%) had an enrollment of 100 participants or fewer. The majority of trials included only female participants. Asia hosted the highest number of clinical trials (46,33.33%), followed by Europe (36,26.09%), Africa (29,21.01%), America (13,9.42%). The majority of trials (61,44.20%) focused on individuals with unexplained recurrent spontaneous abortion (URSA). The predominant intervention types examined in the reviewed studies were drug interventions (49,62.82%), with a notable rise in behavioral intervention trials.

**Conclusion:**

Our research findings suggest that existing research efforts in the realm of RSA are inadequate for the progression of prevention and treatment strategies. The majority of clinical trials have primarily targeted individuals with URSA, with a particular emphasis on drug interventions, notably anticoagulants.

## 1 Introduction

Recurrent spontaneous abortion (RSA), also referred to as recurrent pregnancy loss (RPL), is a significant concern within women’s reproductive health. This condition is linked to both subsequent obstetric complications and enduring health issues, such as cardiovascular disease and mental health implications (1).It is estimated that RSA impacts approximately 1% to 5% of couples attempting to conceive (2). The etiology of RSA is complex and has obvious heterogeneity (3). Numerous risk factors have been identified in association with RSA, including chromosomal abnormalities, infections, endocrinopathies, uterine anomalies, antiphospholipid syndrome (APS), inherited thrombophilia and lifestyle factors (4). Additionally, factors such as improper decidualization (5), and the presence of autoantibodies have also been implicated in RSA (6). Nevertheless, approximately 40-50% of RSA cases are classified as URSA, where the cause of miscarriage remains unknown (7). Furthermore, the lack of consensus regarding the definition of RSA, specifically whether it should be characterized by two or more versus three or more pregnancy losses, complicates the prevention and management of this condition (8).

Various therapeutic interventions have been utilized in the management of RSA in recent decades (9–14). Key treatments are widely recognized to encompass progesterone supplementation, levothyroxine for individuals with hypothyroidism and RSA, and the concurrent administration of heparin and aspirin for patients diagnosed with APS (15). It is important to note that anticoagulant therapy is generally not advised for women with hereditary thrombophilia and RSA (16), except when necessary for the prevention of venous thromboembolism (VTE). However, the existing evidence is inconclusive regarding the efficacy of certain immunomodulatory agents, such as intravenous immunoglobulin (IVIG), intralipid, and granulocyte colony-stimulating factor (G-CSF), in the treatment of URSA. While some studies have reported an improvement in live birth rates (LBR) among patients with URSA following the administration of immunomodulatory agents, others have failed to demonstrate a significant difference (17, 18).Therefore, further high-quality clinical research is imperative to enhance clinical practice and inform strategies for the treatment and prevention of RSA (15).

Clinical studies are the cornerstone of evidence-based medicine, with quality and scope being crucial. The establishment of the ClinicalTrials.gov database was designed to improve transparency and reduce publication bias, ensuring the integrity of study outcomes (19).Currently,ClinicalTrials.gov serves as the largest and most thorough repository of data on active and completed clinical research worldwide (20, 21), featuring registration details for roughly 500,000 studies conducted in more than 200 nations. This registry encompasses a wide array of research pertaining to pharmaceuticals, therapeutic interventions, medical devices, and behavioral strategies, providing comprehensive trial data including objectives, eligibility criteria, design, status, locations, and outcomes (22).The extensive information available on ClinicalTrials.gov offers a distinctive opportunity to comprehend the landscape of clinical trials for specific disease groups or indications.

RSA poses a considerable challenge within the field of reproductive medicine (23). Despite extensive efforts to elucidate its underlying etiologies, effective treatment options remain limited (24). RSA is attracting increasing scholarly attention. A recent Lancet Series published in 2021 critically assessed the existing evidence on miscarriage (15, 25, 26), and an increasing number of clinical studies have been undertaken to investigate the scientific basis of RSA. Consequently, a comprehensive understanding of the current characteristics of RSA clinical trials is essential for improving trial design and identifying neglected areas of research.

Prior research has investigated the attributes of clinical trials listed in the Clinical Trials.gov database across various medical conditions (27–31).However, there is no study focusing on the landscape of RSA clinical trials. The aim of this study is to characterize the various aspects of these trials, such as study design, enrollment, location, and study population in order to provide valuable insights for policy makers, the medical research community and pharmaceutical companies.

## 2 Materials and methods

**Figure 1:**
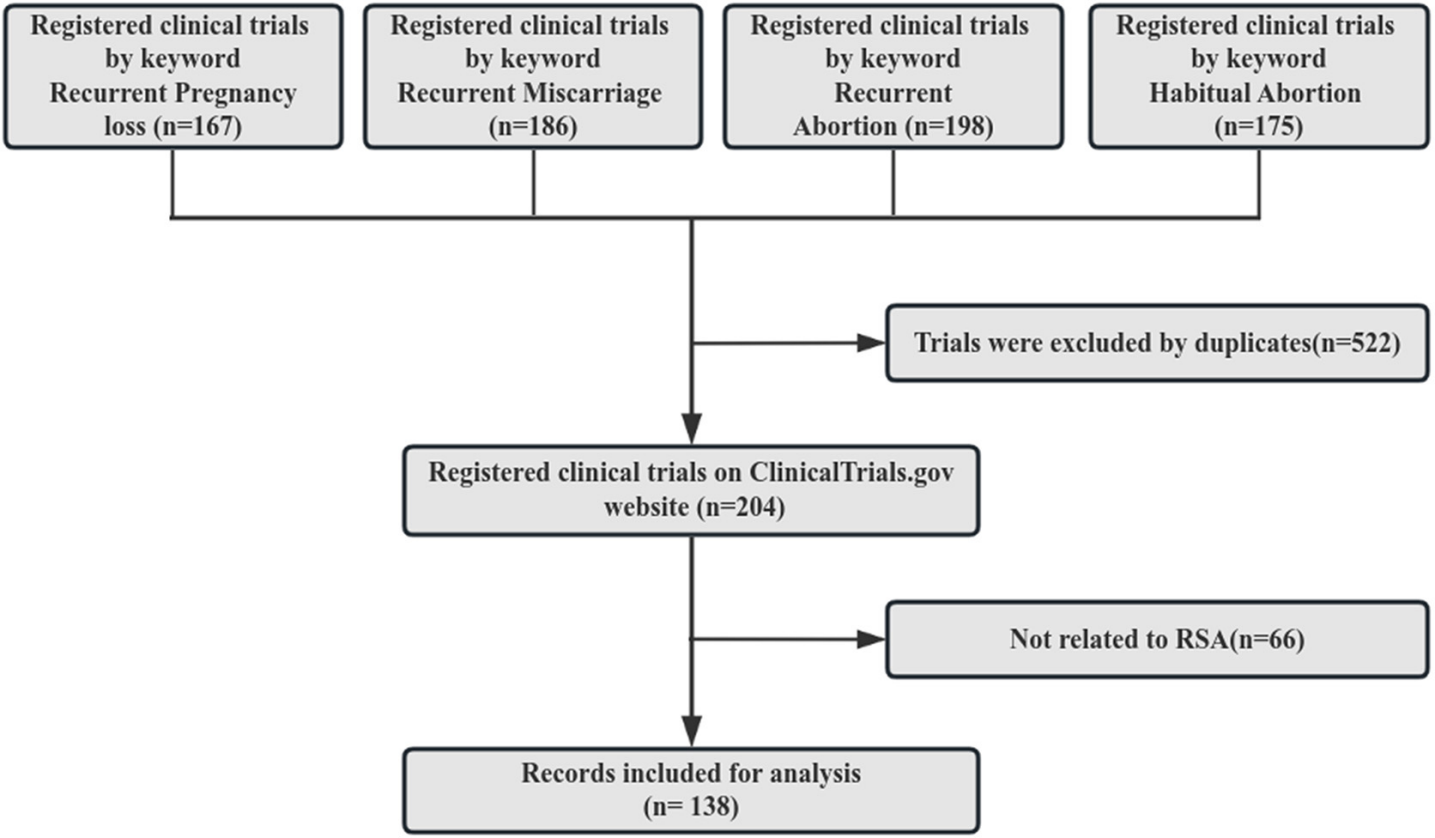
Process of determining the included trials.

As of March 2,2024, a total of 485,171 clinical trials were registered on Clinical Trials.gov. A search was conducted on the Clinical Trials.gov website in March 2024 using the keywords recurrent pregnancy loss, recurrent miscarriage, recurrent abortion, and habitual abortion. Subsequently, the four arms of the identified trials were combined and any duplicates were eliminated. A manual assessment of the study conditions and titles of these trials was carried out to exclude those not relevant to RSA. Ultimately, a total of 138 clinical trials met the inclusion criteria for this study, as depicted in **Figure 1**.

The data collected for analysis included the study title, study type, number of sites, enrollment, funder type, status, start date, locations, condition, trial phases, design, participant gender, and intervention type. A manual review was conducted for study population and number of miscarriages. Study population was defined as individuals eligible for participation in a study. The study was deemed exempt from institutional review board oversight as it solely utilized publicly accessible data devoid of personal identifiers or involvement of human subjects.

## 3 Statistical analysis

Descriptive analyses indicate that categorical variables were represented using both numerical counts and percentages. Data processing and analysis were conducted using Microsoft Office Excel.

## 4 Results

### 4.1 Basic characteristics of registered RSA trials

**Figure 2:**
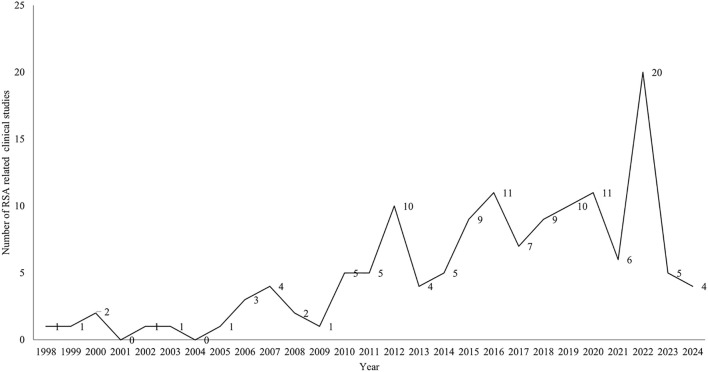
The number of clinical trials by year until Mar 2, 2024.

A total of 138 clinical studies were identified as being related to RSA, consisting of 72 interventional studies (52.17%) and 66 observational studies(47.83%). The majority of trials began in 2010 or later (121,87.68%). The progression of the number of trials over time is illustrated in **Figure 2**. The majority of trials were conducted at single-center facilities (108,78.26%). Of the 138 studies, nearly half had a planned or actual enrollment of 100 patients or fewer(67,48.55%), while only 15 (10.87%) studies enrolled more than 500 patients. The majority of studies included in the analysis were funded by sources categorized as “other” (130,94.20%), with a small percentage funded by industry (6, 4.35%) and NIH/US Federal funding (2,1.45%). Among the 138 studies related to RSA, 60 (43.48%) were completed, 35 (25.36%) had an unknown status, and 15 (10.87%) were actively recruiting participants. The majority of trials included only female participants (130,94.20%). **Table 1** provides an overview of basic clinical trial characteristics.

**Table 1:** Characteristics of RSA related trials registered on Clinical Trials.gov.

Characteristic	n=138, No. (%)
Study type	
Interventional	72(52.17)
Observational	66(47.83)
No. of site	
1	108 (78.26)
≥2	16 (11.59)
Not reported	14 (10.14)
Enrollment of patients	
≤50	27 (19.57)
51-100	40 (28.98)
101-500	55(39.86)
>500	15 (10.87)
Not reported	1 (0.72)
Funding source	
NIH/US Fed	2 (1.45)
Industry	6 (4.35)
Other	130 (94.20)
Recruitment status	
Completed	60(43.48)
Active, not recruiting	5(3.62)
Not yet recruiting	13(9.42)
Recruiting	15(10.87)
Suspended	1(0.73)
Terminated	5(3.62)
Unknown status	35(25.36)
Withdrawn	4(2.90)
Gender of subjects	
All	6(4.35)
Female	130(94.20)
Male	2(1.45)

### 4.2 Geographical distribution and transnational collaborative trials

**Figure 3:**
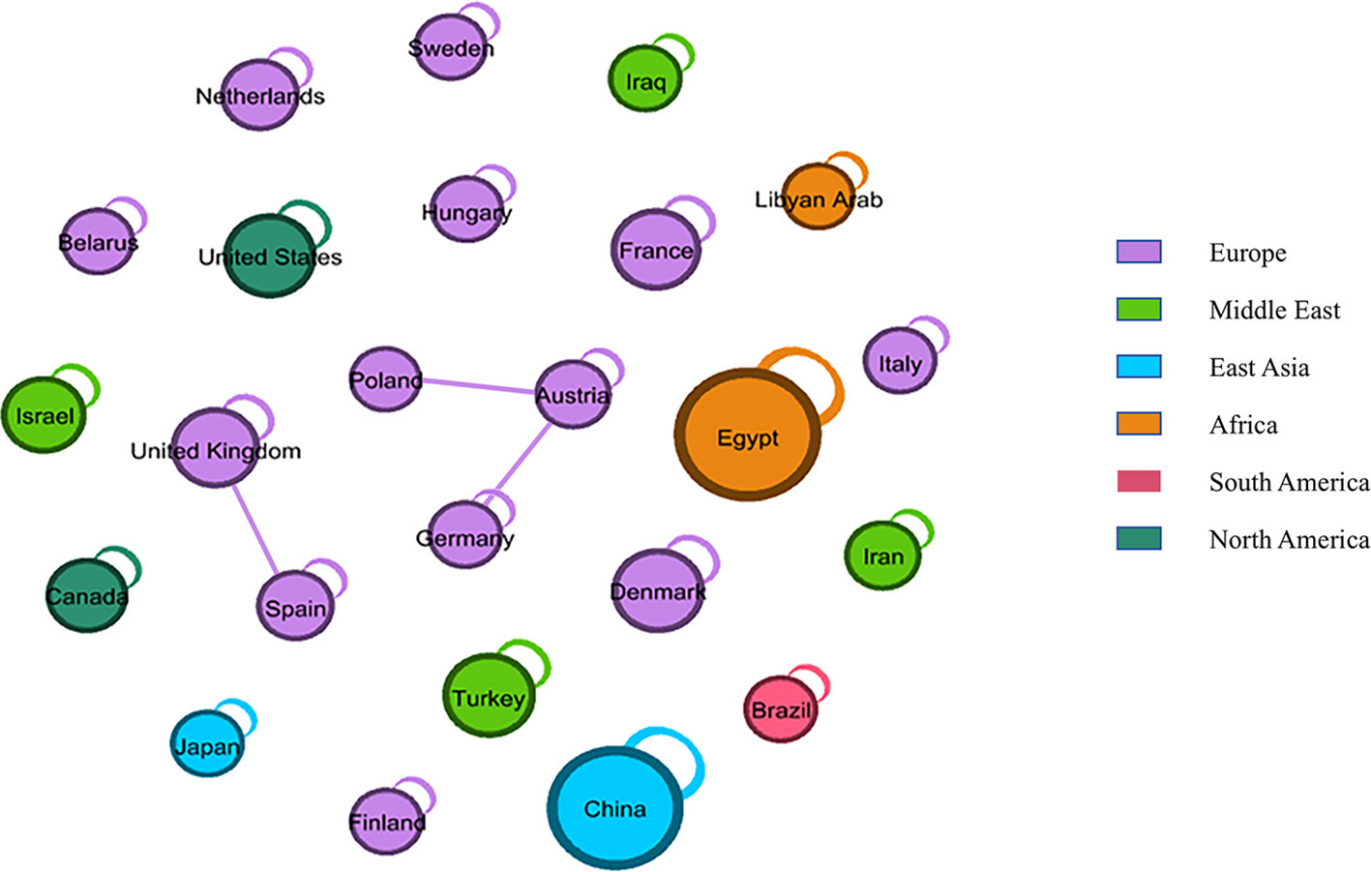
Geographical distribution and transnational collaborative relationships of clinal trials. Each node represents a country, colored by continent. Node size indicates the number of experiments, and lines show country cooperation.

Among the clinical trials examined, 14 did not specify their respective regions. The remaining 124 trials were conducted across various continents. **Figure 3** illustrates the geographical distribution and transnational collaborative relationships of these trials. In this figure, each node represents an individual country, with its color indicating the continent to which the country belongs. The size of the node correlates with the number of trials conducted by that country. Notably, Asia hosted the highest number of clinical trials (46,33.33%), followed by Europe (36,26.09%), Africa (29,21.01%), America (13,9.42%). China hosted nearly one-fifth of all clinical trials (29,21.01%), within three trials in Hong Kong and two in Taiwan. This was followed by Egypt (28,20.29%), Denmark (8,5.80%), Turkey (8,5.80%), and the United States (8,5.80%).

The straight line symbolized the cooperative relationship between countries, indicating that the nodes at both ends of the line corresponded to two countries engaged in international clinical trials. Conversely, nodes not connected to others by a straight line, but instead linked by a curved line pointing to themselves, signified that the country conducted only domestic, rather than transnational, clinical trials. Consequently, European countries demonstrated a transnational collaborative relationship in trials, with the United Kingdom conducting trials with Spain, and Austria collaborating with Poland and Germany.

### 4.3 Study population

**Figure 4:**
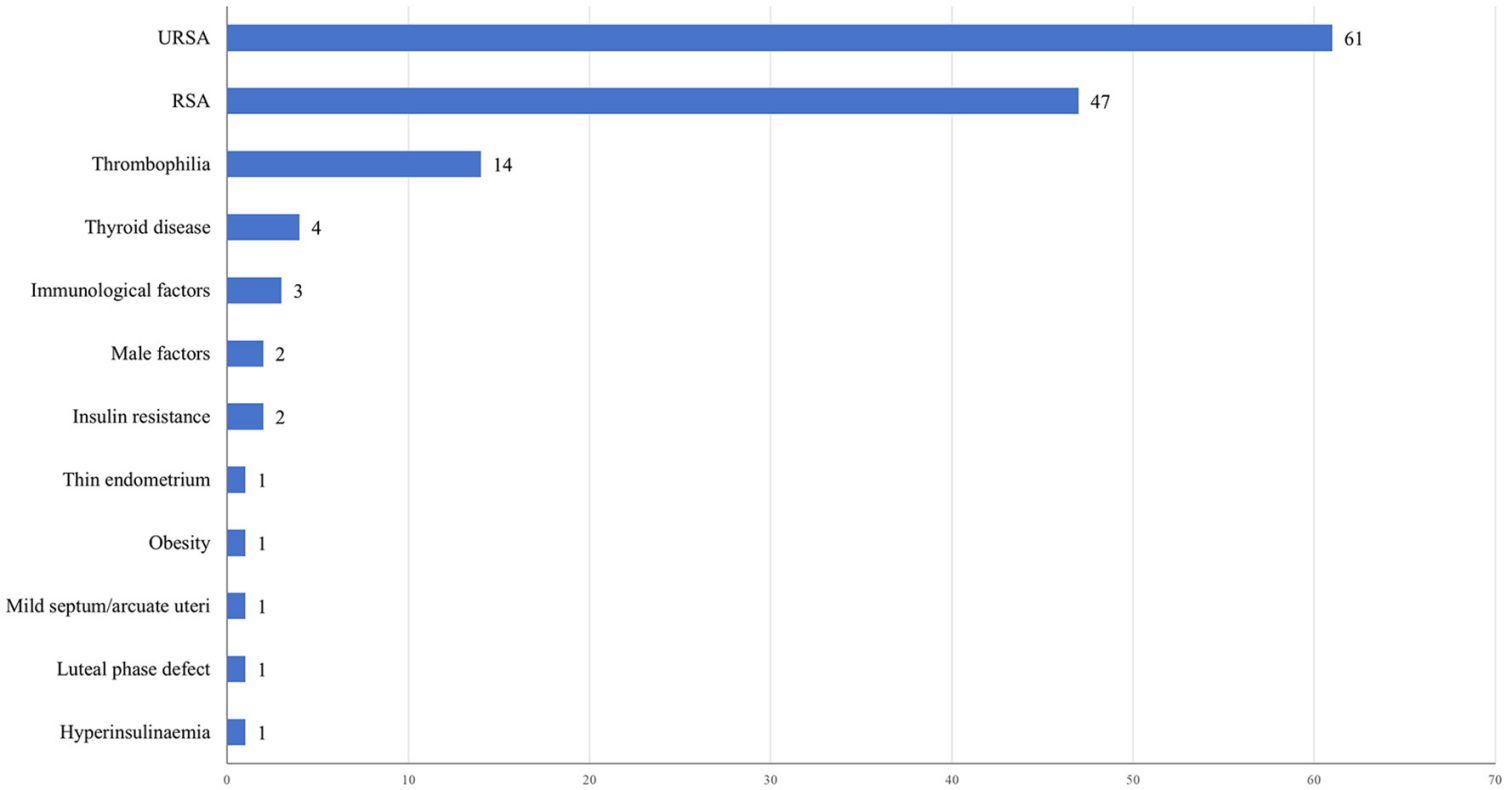
Number of clinical trials by study population.

**Figure 4** illustrates the distribution of clinical trials based on the study population. The majority of trials (61,44.20%) focused on individuals with URSA. Additionally, a considerable proportion of patients with RSA (47,34.06%) were included in trials without specific categorization based on the underlying etiology. Other prevalent study populations encompassed thrombophilia (14,10.14%), thyroid disease (4,2.90%), immunological factors (3,2.17%), male factors (2,1.45%), and insulin resistance (2,1.45%). Out of the 138 studies related to RSA,75 (54.35%) clinical trials involved women who experienced two or more pregnancy losses, while 38 (27.54%) clinical trials included women with three or more losses. Only 50 (36.23%) clinical trials explicitly stated that the miscarriages were consecutive.

### 4.4 Characteristics of interventional studies

A total of 72 interventional studies were included in the analysis, with their characteristics detailed in **Table 2**. The majority of these studies (60,83.33%) utilized a parallel assignment, while a smaller percentage (11,15.28%) employed a single group assignment. Only one study utilized a factorial study design. In terms of allocation strategy, the majority of studies (53, 73.61%) employed a randomized approach, while a smaller percentage (10, 13.89%) used a non-randomized method. Additionally, a further 9 studies (12.50%) did not provide information on the allocation strategy. All interventional trials included in the analysis reported information on masking, with 35 trials (48.61%) utilizing blinding techniques.

**Table 2:** Characteristics of interventional studies.

Characteristic	n=72, No. (%)
Interventional mode	
Parallel	60(83.33)
Single group	11(15.28)
Factorial	1 (1.39)
Allocation	
Randomized	53 (73.61)
Non-randomized	10 (13.89)
Not reported	9 (12.50)
Masking	
Open	37 (51.39)
Single	9 (12.50)
Double	11(15.28)
Triple	3(4.17)
Quadruple	12(16.67)
Intervention type	
Drug	49(62.82)
Procedure	7(8.97)
Biological	2(2.56)
Dietary-supplement	2(2.56)
Diagnostic	1(1.28)
Behavioral	7(8.97)
Device	3(3.85)
Genetic	4(5.13)
Other	3(3.85)
Phase	
Early Phase 1	2(2.78)
Phase 1	3 (4.17)
Phase 1-2	3 (4.17)
Phase 2	10 (13.89)
Phase 2-3	4 (5.56)
Phase 3	11 (15.28)
Phase 4	12 (16.67)
Not reported	27 (37.50)

Percentages may not sum to 100% as rounding.

**Figure 5:**
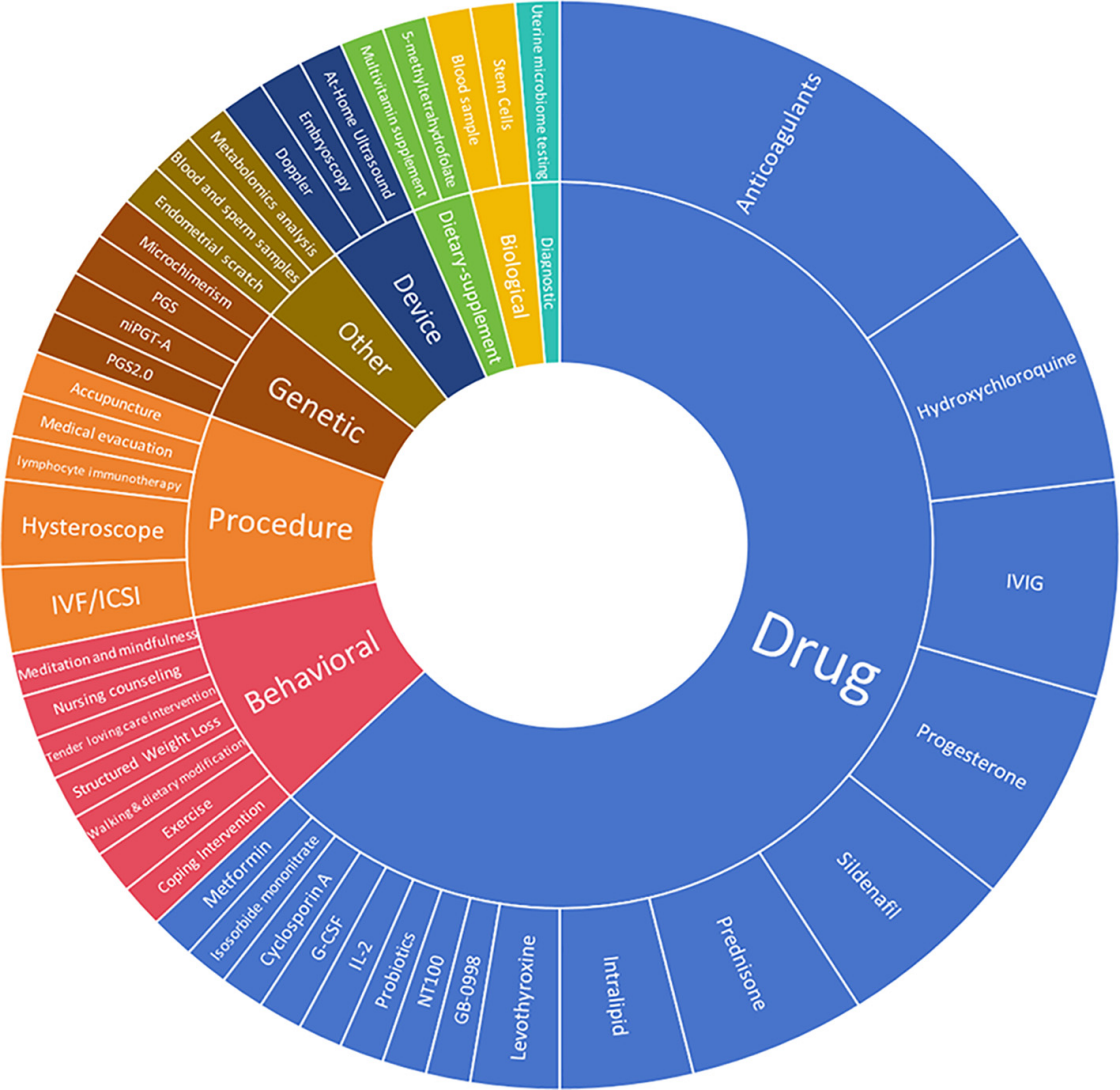
Distribution of clinical trials by intervention type. These included the following interventions. Drug49. Procedure 7(hysteroscope 2,IVF/ICSI 2. lymphocyte immunotherapy 1 acupuncture 1,medical evacuation1). Each specific intervention of behavioral genetic device based, biological, diagnostic, dietary supplement, and other interventions, underwent evaluation in an individual trial.

**Figure 6:**
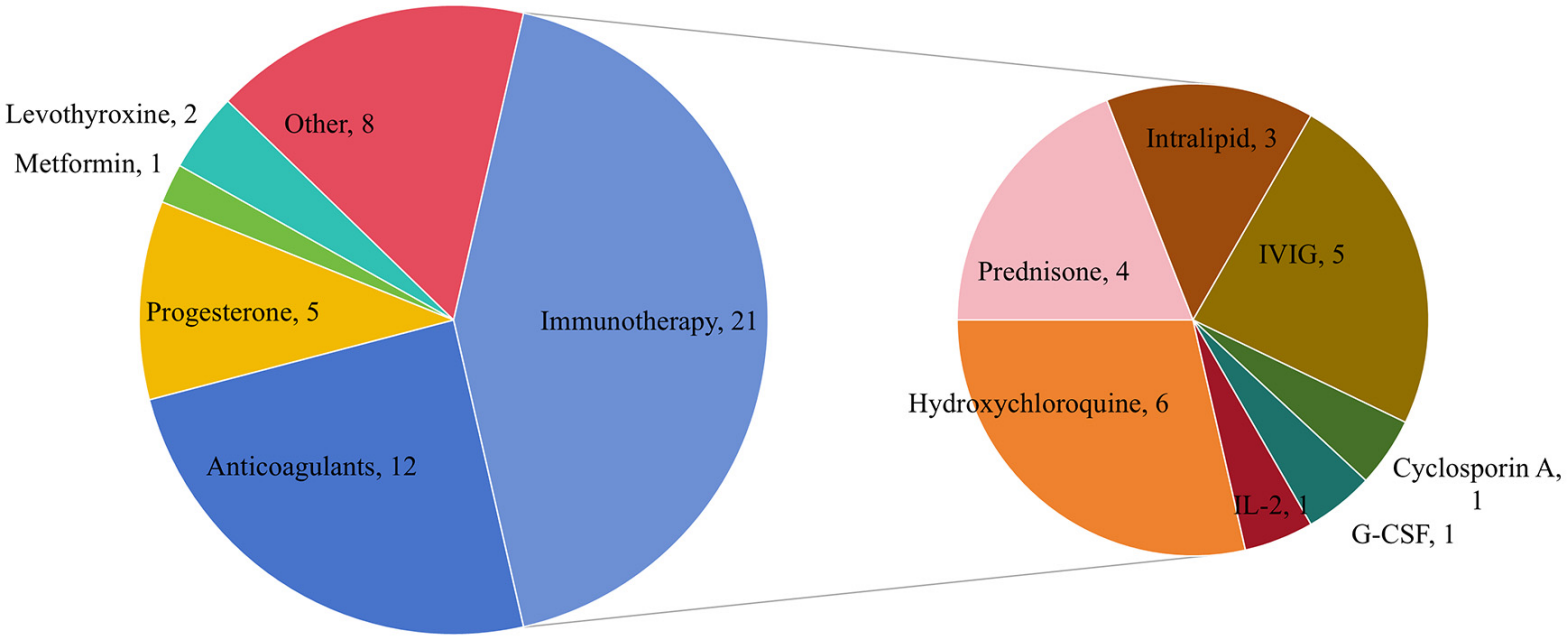
Pie chart showing study drug distribution of registered interventional studies. Other drugs include Sildenafil 4, antibiotics and vaginal probiotics 1, insert vaginal tablet isosorbide mononitrate 1, GB-0998 land NT100 1.

**Figure 7:**
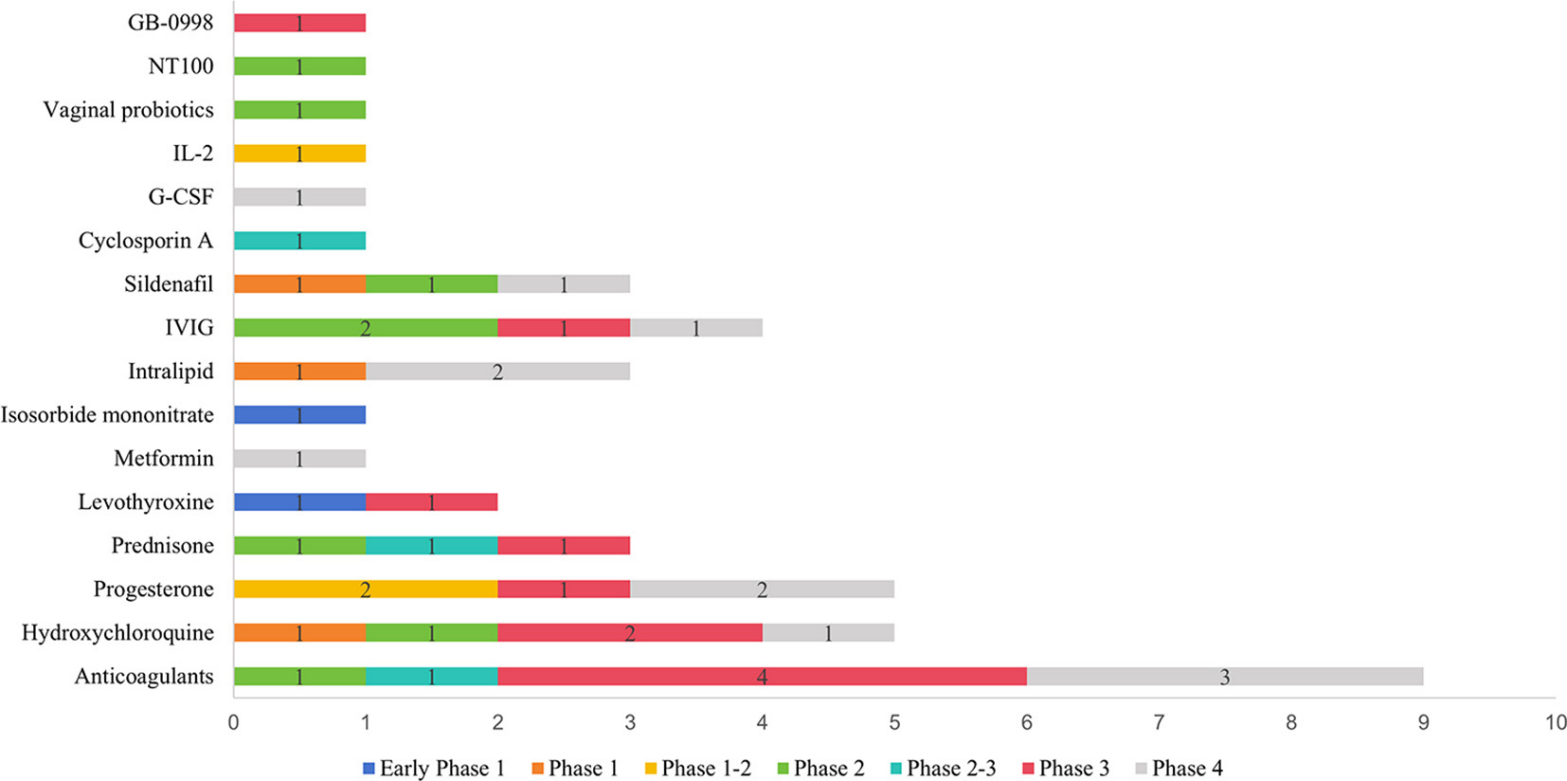
The number of trials in each phase in various drug intervention types.

**Figure 8:**
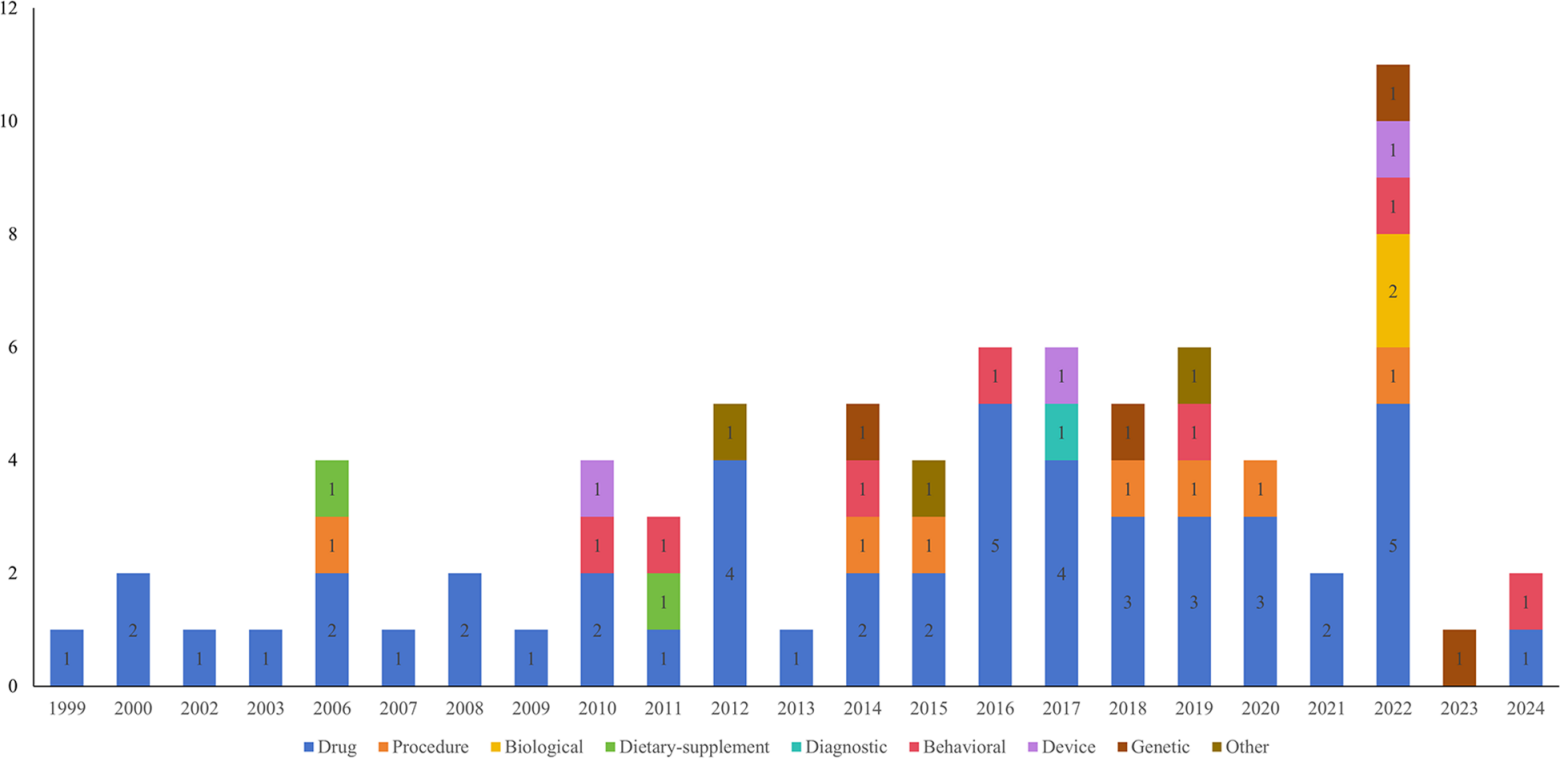
Annual number of RSA clinical trials by intervention type until March 2, 2024.

A total of 78 interventions were identified in various clinical trials. These interventions were categorized as follows: drug (49,62.82%), behavioral interventions (7,8.97%), procedures (7,8.97%), genetic interventions (4,5.13%), devices (3,3.85%), biological interventions (2,2.56%), dietary supplements (2,2.56%), diagnostic interventions (1,1.28%), and other interventions (3,3.85%). **Figure 5** provides a comprehensive breakdown of each intervention type. Within the drug category, anticoagulants were utilized in 12 studies (24.49%), and immunotherapy was employed in 21 studies (42.86%). **Figure 6** presents a pie chart illustrating the distribution of study drugs in registered interventional studies. Notably, approximately one-third of these drug intervention trials did not specify phase data, while slightly more than half were classified as Phases 2-3 or 4 (51.39%). **Figure 7** depicts the number of trials conducted at each phase across various types of drug interventions. Additionally, there is a notable increase in the intervention types from 2010 to 2024, with a particular rise in behavioral intervention trials. **Figure 8** presents the annual number of RSA clinical trials categorized by intervention type.

## 5 Discussion

The objective of this study was to delineate the landscape of registered RSA clinical trials on Clinical Trials.gov, with the intention of establishing a foundation for the advancement of disease prevention and treatment within the realm of RSA.

Our study revealed that RSA related clinical trials comprised a mere 0.03% of all registered trials. The majority of these trials were of a small scale and received funding primarily from sources other than industry or NIH. The limited number of high-quality clinical trials in this area may be attributed to the historically low priority given to miscarriage (32).However, it is crucial to emphasize the necessity for high-quality research in this domain, given the association of RSA with enduring health issues, financial strains, and psychological anguish (15, 25, 26, 32).

The findings of previous meta-analyses did not indicate significant geographical variations in the prevalence of RSA (33). However, the distribution of RSA related clinical trials did not align consistently with regional incidence rates of RSA. Our study revealed that Asia conducted the highest number of registered trials, with China and Egypt being the leading countries in terms of trial quantity. Additionally, Turkey, the United States, and Denmark each hosted eight clinical trials, placing them jointly in the third position. This disparity in trial distribution may be explained by societal and cultural factors that contribute to the stigmatization, discrimination, and ostracism faced by childless women, particularly in low-income regions (15). Furthermore, there is a rising prevalence of RSA in both the United States (34) and China (35). This escalating incidence of RSA in these nations may have led to an increased emphasis on conducting clinical trials in this area.

Men were significantly underrepresented in clinical trials pertaining to RSA, with only 5.80% of such trials including male participants. Despite the fact that half of the embryo’s genetic material is contributed by the male partner, research has predominantly focused on maternal factors such as gene mutations, endocrine disorders, and uterine anatomical abnormalities. Recent studies have suggested that male factors may also be influential in the occurrence of RSA (36–40). One retrospective study has have reported that sperm DNA fragmentation is markedly higher in men experiencing RSA compared to fertile controls (41). However, A prospective cohort study suggest that sperm DNA fragmentation does not appear to be associated with RSA(NCT00447395) (42), highlighting the necessity for further investigation in this domain (43).

Our analysis revealed that the URSA population has been the focus of the majority of studies, as URSA accounts for nearly half of all RSA cases (7).There is a notable concern regarding the inclusion of women with a history of pregnancy losses in clinical trials, with 75 (54.35%) trials specifically enrolling women after two or more losses and 38 (27.54%) trials enrolling women after three or more losses. A significant portion of the studies (50,36.23%) reported that the miscarriages were consecutive. Prioritizing the accurate diagnosis of RSA is crucial in addressing the heightened data heterogeneity arising from variations in study populations, which may be attributed to a lack of consensus on the definition of RSA (44–47).For instance, the Royal College of Obstetricians and Gynecologists (RCOG) defines RSA as a minimum of three first-trimester miscarriages (43),while the European Society of Human Reproduction and Embryology (ESHRE) (3)and Chinese guidelines define it as at least two miscarriages (48).Additionally, only the Chinese guidelines incorporate consecutive miscarriages. Establishing a uniform definition is crucial not only for standardizing protocols, but also for promoting consistency in research and clinical practice (46).

The results of our study suggest that nearly half of clinical trials focusing on RSA are interventional studies. Clinical trials has primarily focused on drug interventions, particularly anticoagulants, which are commonly utilized as a preventive measure for women with APS and are increasingly administered to women with URSA (49).However, ESHRE advise against the use of heparin for women with URSA (3).This recommendation is supported by evidence from two multi-center randomized trials, which found no significant benefit of heparin compared to placebo or multivitamin supplementation(NCT00740545,NCT00400387) (50, 51).Additionally, several trials have investigated the efficacy of new medications, such as IVIG, intralipid, and G-CSF, in addressing the clinical needs for URSA treatment. A high-quality randomized controlled trial (RCT) identified that IVIG administration significantly enhances LBR in women experiencing four or more unexplained pregnancy losses (NCT02184741) (52). Based on these findings, ESHRE conditionally recommends the early administration of repeated high-dose IVIG therapy in pregnant women with URSA (3). However, the efficacy of G-CSF remains inconclusive, as evidenced by conflicting outcomes from two clinical trials involving women with URSA. In one RCT, G-CSF was associated with an improved LBR compared with placebo(NCT00772122) (53). Conversely, in a larger RCT, G-CSF did not demonstrate any benefit(NCT02156063) (54).Given the high quality of the latter trial, ESHRE considers its findings to supersede earlier reports and does not recommend the use of G-CSF in patients with URSA (3). In addition, ESHRE does not recommend intralipid therapy for URSA due to insufficient evidence (3).

While clinical studies on RSA drugs constitute the majority of intervention research, novel treatment approaches, particularly behavioral interventions, are continually being developed. At present, seven pertinent studies are registered on ClinicalTrials.gov, with three having reached completion (NCT02989220, NCT04361747, NCT03023137), however, their findings have yet to be disclosed. Notably, some research has indicated a correlation between stress, obesity, and RSA (25, 55–60).Both ESHRE and RCOG advocate for additional research to advance psychological support and lifestyle modifications for RSA women and their partners (3, 43).

Our study has some limitations. Firstly, it should be noted that Clinical Trials.gov does not encompass all clinical trials related to RSA conducted globally, as a portion of trials are registered in alternative databases such as the Chinese clinical trial registry or European union clinical trial Registry. Although we considered incorporating additional registries into our analysis, doing so could potentially compromise the level of detail and accuracy afforded by utilizing a single data source. The data fields across different registries are not uniform, either in terms of their existence or their coding, and the reporting standards of some international registries may not be applicable to ClinicalTrials.gov (61). Secondly, certain trials may have omitted crucial elements or keywords during registration. Lastly, misclassification may potentially result in erroneous conclusions being drawn from the data.

## 6 Conclusion

This research offers a comprehensive overview of global clinical trials related to RSA. Our results indicate that the research activity of RSA is inadequate for the progression of prevention and treatment strategies. There is a pressing need for high-quality research and further investigation into the roles of psychological support and male factors in RSA.

## Data Availability

The original contributions presented in the study are included in the article/supplementary material. Further inquiries can be directed to the corresponding author.
